# Research on Laser Marking Speed Optimization by Using Genetic Algorithm

**DOI:** 10.1371/journal.pone.0126141

**Published:** 2015-05-08

**Authors:** Dongyun Wang, Qiwei Yu, Yu Zhang

**Affiliations:** The Engineering School, Zhejiang Normal University, Zhejiang, China; University of Catania, ITALY

## Abstract

Laser Marking Machine is the most common coding equipment on product packaging lines. However, the speed of laser marking has become a bottleneck of production. In order to remove this bottleneck, a new method based on a genetic algorithm is designed. On the basis of this algorithm, a controller was designed and simulations and experiments were performed. The results show that using this algorithm could effectively improve laser marking efficiency by 25%.

## Introduction

Laser marking machines (LMMs) have become the most common coding equipment on product packaging lines. These machines are commonly used for their tamper-resistance and environmentally friendly characteristics. However, the speed of LMMs cannot keep up with the speed of the pipeline and affect production. Currently, studies of methods for improving marking efficiency generally focus on Laser Travelling Path Optimization (LTPO). Qiwei Yu and Dongyun Wang [1] [2] changed the LTP when a basic marking unit was completed. These methods could help improve the product line’s speed, which is usually limited by the size of marking area. However, the efficiency of the basic marking unit could not be improved. Xin Sun [3] transformed the contour’s cutting sequence problem into a TSP problem, which resulted in a significant improvement in coding of closed contour lines. It could help improve the laser’s travel efficiency to some extent. Nini Li[4] extracted the nodes from the contour, then optimized the path nodes using a local search optimization algorithm, and eventually obtained an approximate optimal solution. To further improve laser marking efficiency, a new method based on GA is proposed in this paper.

## Materials and Methods

### Principles of LMM and description of LTPO problem

**Fig 1:**
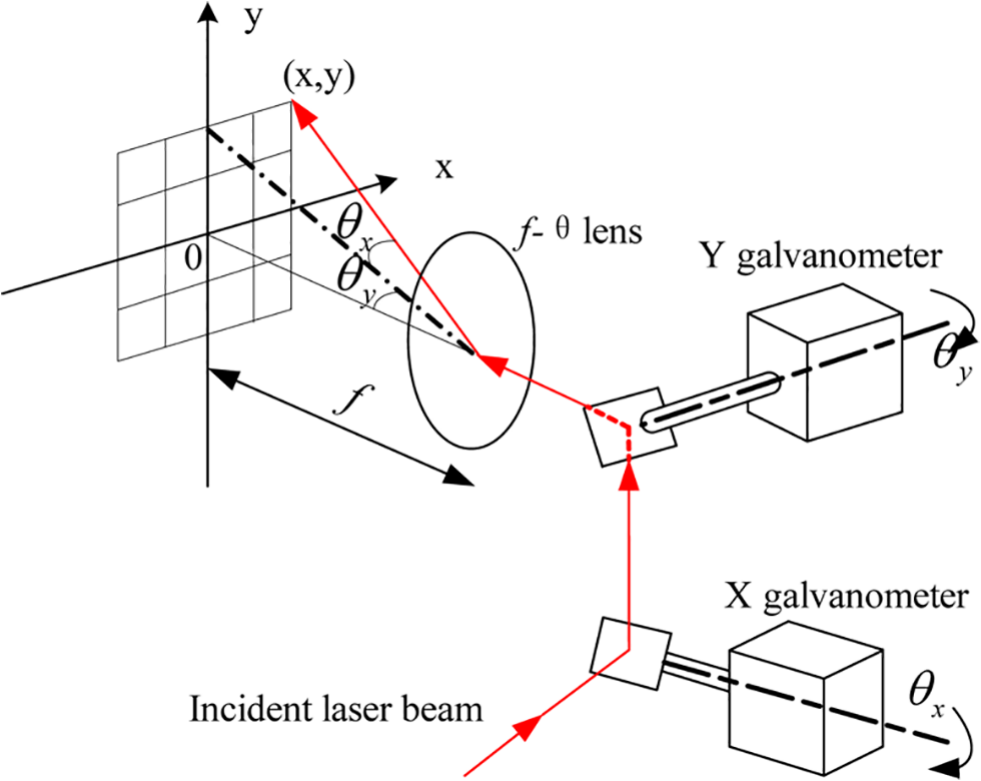
Schematic diagram of the two-dimensional laser marking system.

In galvanometric scanning system, a laser beam is deflected by two mirrors, and then focused by an f-theta lens. At last, the focused beam is projected on the marking plane [5]. Fig 1 shows the general principles of LMM. The position of the marking point is determined by the rotating angles of X, Y galvanometers [6]. If the point should be marked, the laser will be turned on. Otherwise it will be shut down. Through combination control of laser power switch and galvanometer angles, any two-dimension contents could be marked.

There are two factors that affect marking speed, one is the response frequency of galvanometer and another is the length of LTP. Marking contents are composed of points, lines and closed contours. The length of the LPT is determined by the sequence that the laser travels through the content. As all points must be marked exactly once and the laser has to return back to its initial position, the target of LPTO is to find the shortest path.

### LTPO by using genetic algorithm

In the computer science field of artificial intelligence, a genetic algorithm (GA) is a search heuristic that mimics the principle of natural selection and “survival of the fittest” [7].This heuristic is routinely used to generate useful solutions to optimization and search problems [8]. In a genetic algorithm, a population of candidate solutions (called individuals or chromosomes) to an optimization problem is evolved toward better solutions. Each candidate solution has a set of properties which can be mutated and altered [9][10]. GA is great for finding solutions to complex search problems. They're often used in fields such as engineering to create incredibly high quality products. It has the ability to search through a huge combination of parameters to find the best match [11]. The basic steps of GA are shown below [7].

Step1: Generate an initial population
Step2: Evaluate fitness of individuals in the population
Step3: Repeat
  ① Select individuals from the population to be parents
  ② Recombine parents to produce children
  ③ Mutate the children
  ④ Evaluate fitness of the children
  ⑤ Replace some or all of the population by the children

Until

Decide to stop whereupon report the best solution encountered

### Gene and chromosome representation for LTPO

Various representation methods are used in GA, such as binary representation, path representation, matrix representation, adjacency representation, ordinal representation [11]. The LTPO problem is to find a shortest path for a given set of marking content. So it is natural to using path representation. The chromosome corresponding to a LPT is an array of n integers which is a permutation of (1, 2,…, *n*), where an entry *i* in position *j* indicates that point *i* is visited in the *j* th time instant. The first step in using a genetic algorithm is to set up the relationship between the solution Space and the chromosome’s coding space.

**Fig 2:**
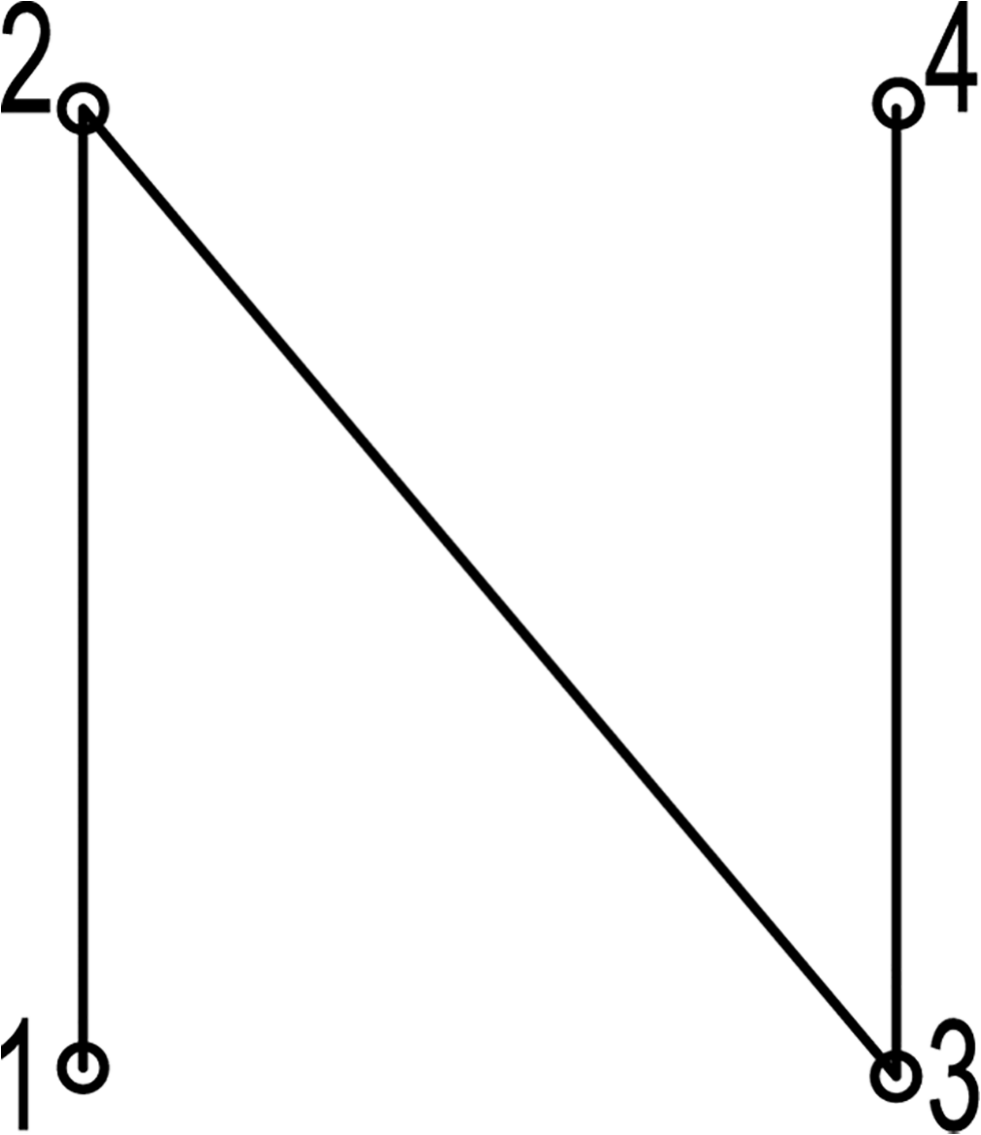
The process of encoding a chromosome.

A gene refers to a point to be marked and gene encoding which is a part of chromosome encoding, refers to describing the marking points with natural numbers. Therefore chromosome P(1,2,3,4) expresses a path that the laser will take when it marks an object, as shown in Fig 2.

**Fig 3:**
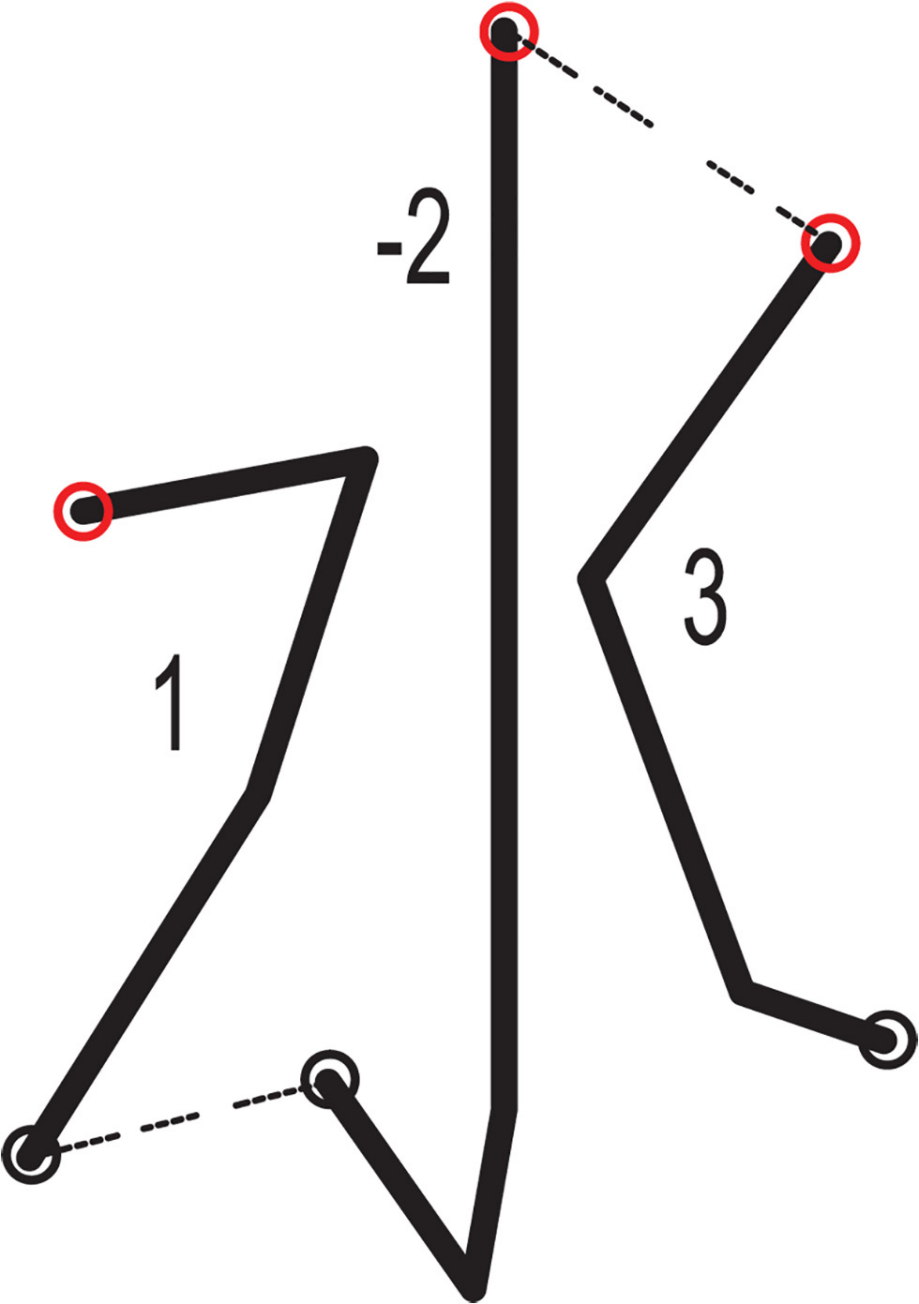
Encoding a closed contour into a Chromosome.

Each contour’s start point and end point are considered the index points of the closed contour in the gene encoding. Now each gene expresses a closed contour composed of a group of points, but stepping along the closed contour should be conducted when marking each indexed point. As described in Fig 3, chromosome encoding is to compose a gene sequence for the marking contents.

**Fig 4:**
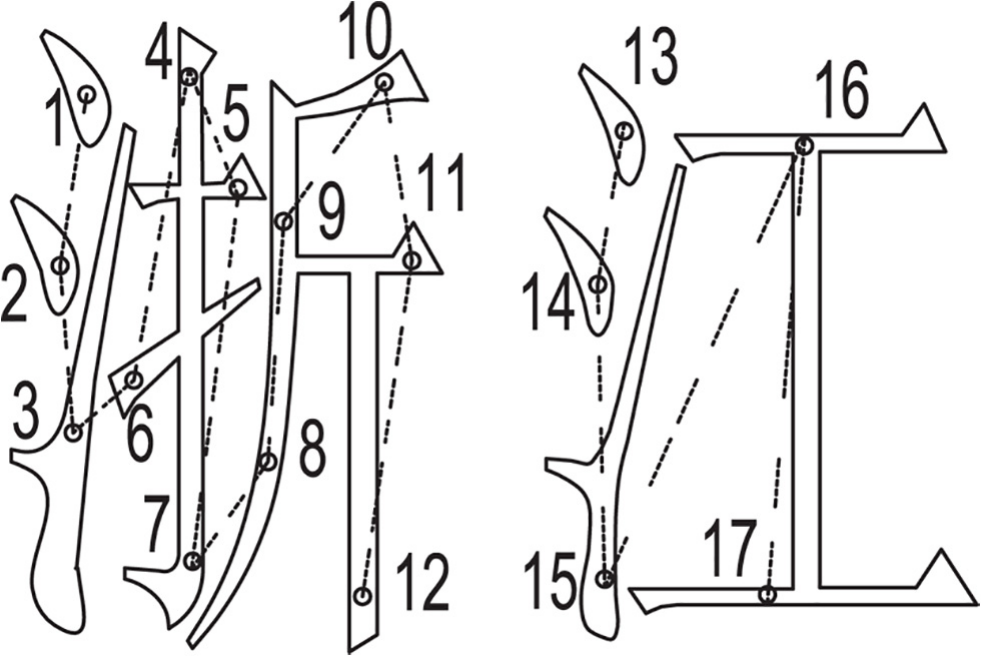
Encoding an open line into a chromosome.

Fig 4 shows the shape of an open line which consists of lines connected end to end. Each line has start and end points between which is a continuous path. The marking process could select to begin from either the start point or the end point. Therefore, positive and negative signs are introduced indicate the marking direction. A positive sign means that a line is processed from its start point to end point, while a negative sign means the contrary. A description of each open line by the natural number that expresses the marking sequence of the strokes, in this case the relationship between the solution space of the LTPO and the positive and negative integers representing the path in coding space is set up.

### The fitness function

Let {1,2,3,4 … n} be the labels of the points and D = d[i,j] be an n × n cost matrix where d[i,j] denotes the distance from i to j. The LTPO problem is to find the shortest path for the laser to travel through all the points. The length of the path is given by

$$\text{D}\left(\text{n}\right)=\overset{n-1}{\underset{0}{\sum }}d\left[i,i+1\right]+d\left[n,1\right]$$ (1)

The objective is to find a permutation of the n points, which has the shortest path.

As the coordinates for point i are given as (*x*_*i*_,*y*_*i*_), the length of the path can be further calculate as

$$\text{D}\left(\text{n}\right)=\overset{n-1}{\underset{0}{\sum }}\sqrt{{\left(x_{i}-x_{i-1}\right)}^{2}+{\left(y_{i}-y_{i-1}\right)}^{2}}+\sqrt{{\left(x_{n}-x_{1}\right)}^{2}+{\left(y_{n}-y_{1}\right)}^{2}}$$ (2)

So the fitness function can be given by

$$\text{f}\left(\text{n}\right)=\frac{1}{\text{D}\left(\text{n}\right)}$$ (3)

### Initializing the population

Many individual solutions are randomly generated to form an initial population. The population size depends on the nature of the problem, but the initial population typically contains several hundreds or thousands of possible solutions. Traditionally, this population is generated randomly, allowing the entire range of possible solutions (the search space). In LTPO, the size of the population is determined by the complexity of the marking contents. To keep the group’s diversity and calculating complexity, the initial population is randomly generated.

### The genetic operators

Genetic operators include selection, crossover and mutation. The genetic operators could ensure better convergence for GA.

The basic part of selection process is to stochastically select from one generation to create the basis of the next generation. The requirement is that the fittest individuals have a greater chance of survival than weaker ones. This replicates nature in that fitter individuals will tend to have a better probability of survival and will go forward to form the mating pool for the next generation. Weaker individuals are not without a chance. In nature such individuals may have genetic coding that may prove useful to future generations. There are a few different selection methods such has roulette wheel, tournament, linear normalization [11]. Here, the roulette wheel selection is used. And the probability of each individual is based on the fitness value. It is given by

$$\text{P}\left[\text{i}\right]=\frac{f\left[i\right]}{\sum_{j=1}^{N}f\left[j\right]}$$ (4)

**Fig 5:**
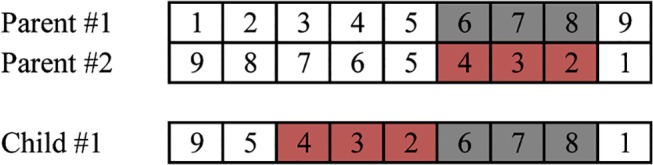
Example of order crossover function.

As the LTPO is a permutation problem, it is natural to encode a path by enumerating the point indices in order. Here, the position of a point is not fixed and only the sequence is meaningful. A valid solution would need to represent a path where every point is included at exactly once. This would mean adjusting the crossover function so it doesn't just add a random point to the path, possibility causing a duplicate. In order to avoid invalidation, the order crossover approach was used. It has been found to be one of the best in terms of quality and speed [11][12]. Fig 5 indicates one example of order crossover. In real application, 2 children were produced.

As for mutation operator, the simple inversion mutation (SIM) approach is used. It has been found effective in TSP solution[12].In SIM, we only need to select two objects at random then simply swap their positions. This could also avoid invalidation.

**Fig 6:**
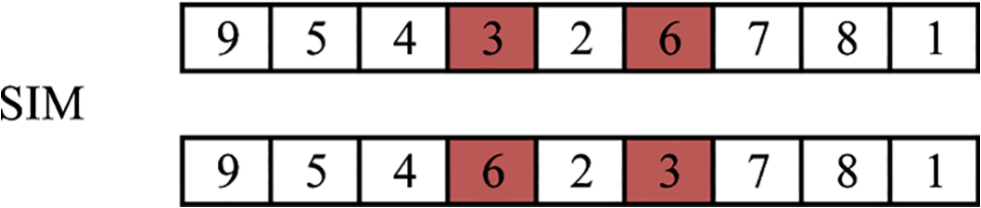
Example of SIM function.

Fig **6**shows an example of SIM function.

### Setting the parameters

Crossover probability (CP) and mutation probability (MP) should be set before optimization process. CP indicates a ratio of how many couples will be picked for mating. The higher of CP would increase the possibility that an excellent individual would be damaged. But a too small one may cause the GA stagnant. In our application the CP was set to 0.8.

MP determines how often will be parts of chromosome mutated. If there is no mutation, offspring is taken after crossover without any change. If mutation is performed, part of chromosome is changed. Mutation is made to prevent falling GA into local extreme, but it should not occur very often. Otherwise GA will in fact change to random search. Here the MP is set to 0.01.

### Simulations and experiments

An open line model is universal for LTPs. A point is the special case that occurs when the line length is zero and start and end points coincide, while a closed contour occurs when the line length is greater than zero and the two points coincide. The algorithm takes the start point as the representative point, while marking to this point, related strokes are also required to be marked. A smaller sum of the length of the paths between the representative points corresponds with a better marking efficiency.

**Fig 7:**
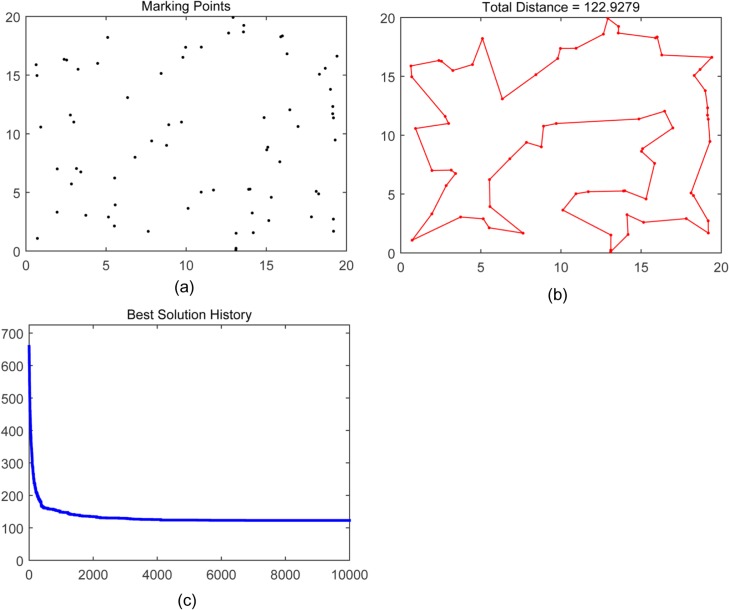
The optimization process by using GA.

The genetic algorithm for LTPO was implemented using the M language and simulated using Matlab. In the simulation, the 70 points shown in Fig 7 (A) were selected for marking. Once started, the GA continued its calculations. And the optimization process was recorded. Fig 7 (B) shows the recommended path for the LTP which has a total length of 122.9279. Fig 7(C) shows the iterative optimization process by recording the route length.

**Fig 8:**
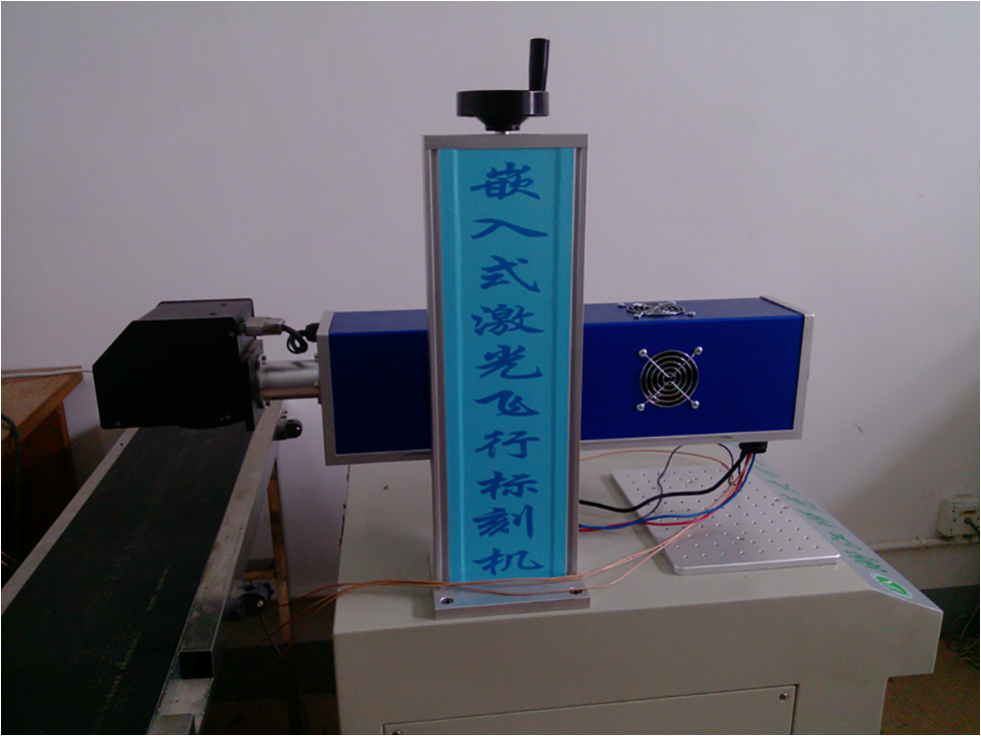
The LMM for experimental study.

To further verify the proposed algorithm, experimental studies were performed. The key components of the LMM are listed in Table 1. Fig 8 shows the picture of the test bench.

**Table 1:** The parameters of the key components of the LMM.

Components	Model	Key parameters
Laser generator	Synrad 48–1 CO2 laser generator	Power:10W
		Wave length: 10.6um
Galvanometer	TS8203 product by Beijing Century Sunny Technology Co. Ltd	Supply volt:±24V±10%
		Input volt: -10V-+10V
Controller	Self-developed	ARM9+Linux2.4+FPGA

**Fig 9:**
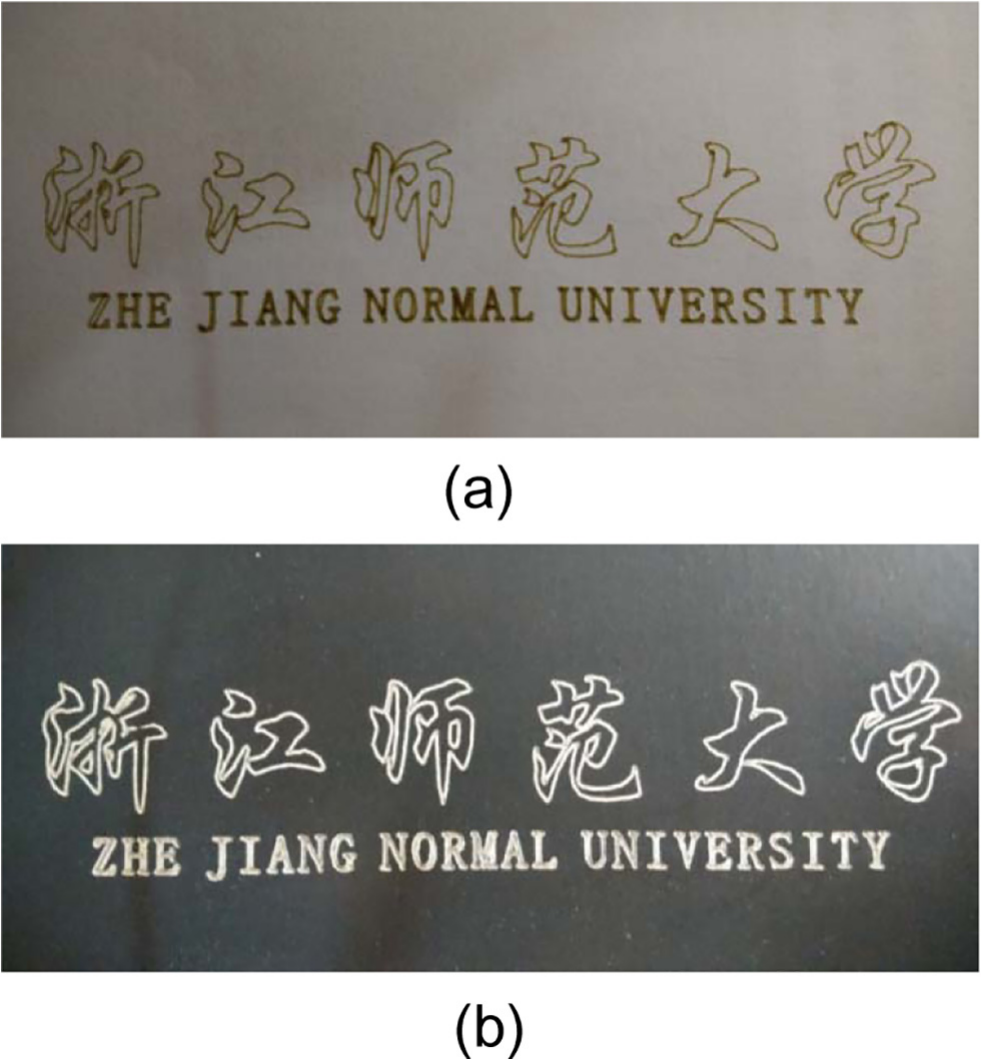
The results of marking using two LTP methods. (a)Marked using traditonal LPT methods. (b) Marked using GA.

To mark the contents of Zhejiang Normal University in both Chinese and English fonts, the first point of each stroke was taken as a representative marking point. The experiment data was recorded in Table 2.The total path length was 8161.67 when the traditional LTP method described in reference [1] was used, and the length of the path found by the GA was 5876.42, which represents a reduction of 28%. Then, the contents were marked twice using the two LPT control methods. Fig 9 shows the marking results. The time required to mark the same content was 3.1s and 2.32s. The efficiency has been improved by approximately 25%.

**Table 2:** A comparison of the traditional LPT method and the GA method.

	Traditional LPT methods	GA methods	improvement
Path length(Normalized)	8161.67	5876.42	28%
Marking time used(S)	3.1	2.32	25%

## Conclusion

In this paper, a new laser traveling path optimization method using GA was introduced and discussed. It was designed by M language and implemented on the test bench. The initial population was chosen randomly. The selection approach was roulette wheel selection. And the CP and MP were set to 0.8 and 0.01 respectively. Simulations and experiments have shown that the new method could achieve more than 25% over the traditional methods. We can conclude that more complex marking contents will produce better optimization effects.
